# Enhanced Response of Metformin towards the Cancer Cells due to Synergism with Multi-walled Carbon Nanotubes in Photothermal Therapy

**DOI:** 10.1038/s41598-017-01118-3

**Published:** 2017-04-21

**Authors:** Sweejiang Yoo, Jin Hou, Wenhui Yi, Yingchun Li, Weiping Chen, Lingjie Meng, Jinhai Si, Xun Hou

**Affiliations:** 1 Key Laboratory for Physical Electronics and Devices of the Ministry of Education & Key Laboratory for Information Photonic Technology of ShaanXi Province, School of Information and Electronics Engineering, Xi’an Jiaotong University, Xi’an, 710049 China; 2 Department of Pharmacology, School of Basic Medical Sciences, Xi’an Medical University, Xi’an, 710021 China; 3 Department of Applied Chemistry, School of Science, Xi’an Jiaotong University, Xian, 710049 China

## Abstract

Converging evidence from laboratory models pointed that the widely used antidiabetic drug metformin has direct effects on cancer cells. Thus far, relatively little attention has been addressed to the drug exposures used experimentally relative to those achievable clinically. Here, we demonstrated that metformin loaded on carbon nanotubes under near-infrared (NIR) irradiation led to the remarkably enhancement in response towards cancer cells. The dose of metformin has reduced to only 1/280 of typical doses in monotherapy (35: 10 000–30 000 µM) where the realization of metformin in conventional antidiabetic doses for cancer therapies becomes possible. The heat generated from carbon nanotubes upon NIR irradiation has mediated a strong and highly localized hyperthermia-like condition that facilitated the enhancement. Our work highlight the promise of using highly localized heating from carbon nanotubes to intensify the efficacy of metformin for potential cancer therapies.

## Introduction

A series of *in vitro* studies and xenograft models have evidenced that metformin has direct action on cancer cell growth and proliferation, where the effects are observed in a wide range of cancer cell lines, including breasts, colon, glioma, ovarian and endometrial^1–4^. Several studies have reported that the combination of metformin with chemotherapeutic drugs leads to an increased sensitivity to chemotherapy or a decreased drug dose for variety of cancer cell lines^5, 6^. The very promising preclinical data have drawn much attention, so metformin is proposed as an anticancer agent in nondiabetic patients. However, people have deep concerns on its excessively high dose for anti-cancer therapy in comparison to that for type 2 diabetes mellitus(T2DM) treatment^2, 7^. It has been demonstrated that the anticancer action in *in vitro* studies only prominent under the condition of high doses, typically from 10–30 mM, which is 3 orders higher than those in T2DM treatment (maximal plasma concentration approximately 10 to 30 µM)^1, 2, 4, 5, 8–15^. Such high doses of metformin cause fatal adverse effects, particularly metformin-associated lactic acidosis (MALA)^2, 11, 12, 16, 17^. Thus, it is of great importance to further enhance the efficacy of metformin in anticancer treatment so as to lower the dose to avoid the fatal adverse effects. It has been widely accepted by the community that the current research goal is to evaluate metformin itself at conventional antidiabetic doses for possible use in oncology, particularly for indications that may require long-term administration, where its extensive safety record is of paramount importance^18^.

Intriguingly, the landmark study by Hirsch *et al*. has revealed that low doses of metformin (0.1 or 0.3 mM, ~10-fold higher than plasma concentrations of metformin in diabetic patients) selectively kill cancer stem cells (CSCs)^14, 19^. CSCs are well resistant to chemotherapy and thus responsible for cancer relapse. Recently, Lee *et al*. have reported that the responses of breast cancer cell to metformin are intensified under hyperthermia condition although the metformin concentration is only 30 μM which is as low as the plasma concentration of metformin in type 2 diabetes patients under metformin treatment^8^. More recently, Eikawa *et al*. demonstrated that metformin exerts an immune-mediated antitumor effect by protect CD^8+^ tumor-infiltrating lymphocytes (TILs) from apoptosis and inevitable functional exhaustion in the tumor microenvironment, which eventually results in tumor growth inhibition^20^. The direct effect on CD^8+^ TILs are observable even at a physiologically relevant low concentration of 10 μM.

Following investigation of metformin at conventional antidiabetic doses for the antineoplastic activity, either in combined with other therapeutic method (hyperthermia/radiotherapy/chemotherapy) or prolong treatment time, indicated possible alternative to the aggressive dosing^8, 13, 21, 22^. As inspired by Lee’s work, we propose that the loading of metformin onto multi-walled carbon nanotubes (MWNTs-Met/PEG) under NIR irradiation would maximize the responses of cancer cells to metformin and reduce the dose accordingly. The photothermal and selective internalization of carbon nanotube inside the cells provides the highly localized heating and the function of targeting drug delivery^23, 24^. Taking advantages of above, we attempted to further intensify the cytotoxicity of metformin towards cancer cells in the photothermal conditions in comparison to that in the conventional hyperthermia conditions.

## Results and Discussion

**Figure 1:**
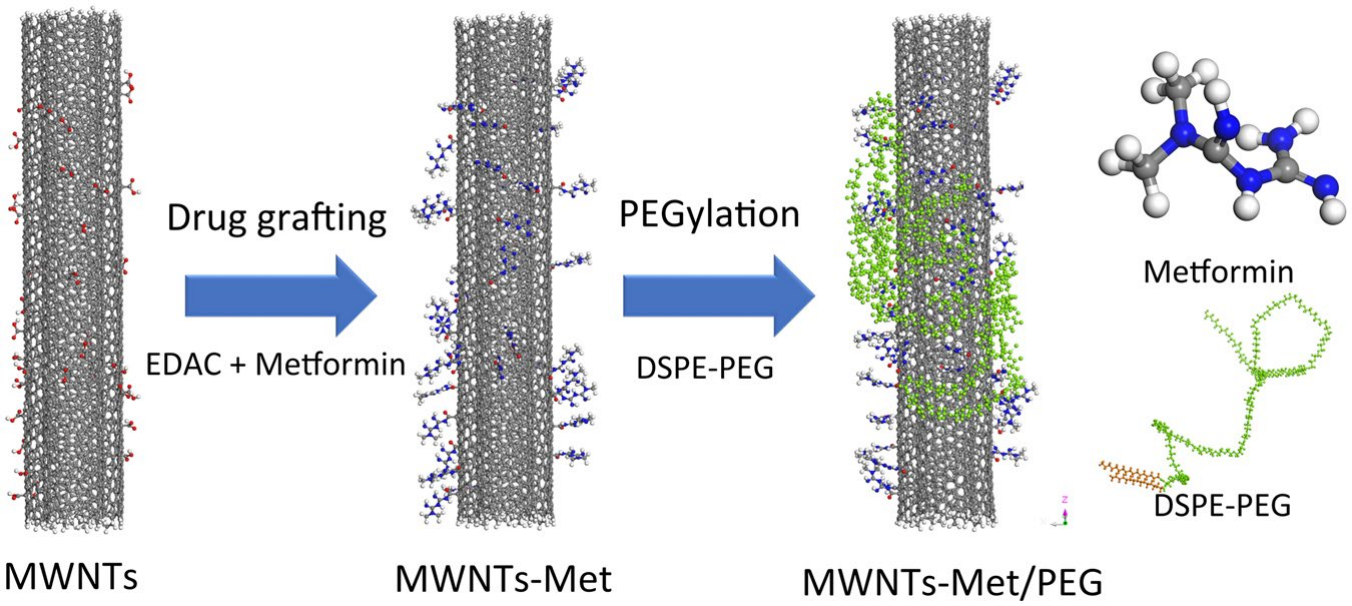
Illustration diagram of MWNTs-Met/PEG synthesis.

**Figure 2:**
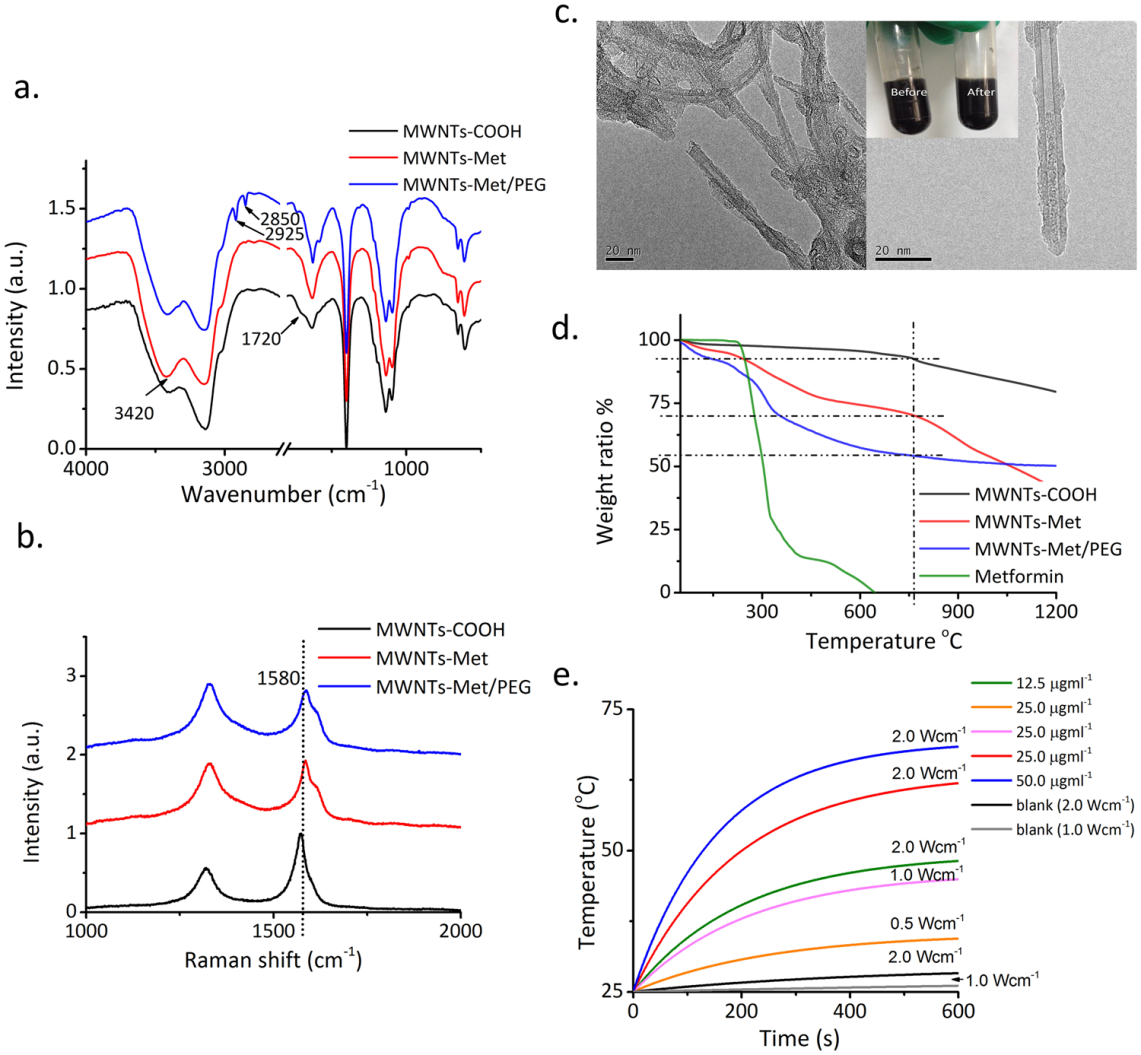
(**a**) FTIR spectra of MWNTs-COOH, MWNTs-Met and MWNTs-Met/PEG. (**b**) The Raman spectra of MWNTs-COOH, MWNTs-Met and MWNTs-Met/PEG. (**c**) TEM images of MWNTs-Met/PEG. The inset is the photographs of MWNTs-Met/PEG before and after 3000 rpm centrifugations. (**d**) The TGA curves of metformin, MWNTs-COOH, MWNTs-Met and MWNTs-Met/PEG. (**e**) Photothermal response of MWNTs-Met/PEG under various concentration (µg·mL^−1^) and NIR power density. The photothermal response is concentration and power dependent. The photothermal response curves were fitted with exponential function and R^2^ > 0.99.

The preparation of MWNTs-Met/PEG is illustrated in Fig. 1. The chemical structure of MWNTs-Met/PEG was characterized with FTIR spectra as shown in Fig. 2a. The MWNT-COOH has a distinctive peak at 3140 cm^−1^ and a shoulder peak at 1720 cm^−1^ which are attributed to the O-H stretching and the C=O stretching of the carboxyl group, respectively. The carboxyl groups were introduced by the acidic oxidation treatment for linkage purpose. MWNTs-Met has shown a diminishing C=O shoulder peak of the carboxyl group due to the formation of amide bond. The signature peak of C=N stretching at 1630 cm^−1^ and N-H stretching at 3420 cm^−1^ become intensified, indicating the successful grafting of metformin. For MWNTs-Met/PEG, the PEG distinctive peaks are clearly observed at 2850 cm^−1^ and 2925 cm^−1^ which are arising from the C-H stretching. PEG chains helically wind around the tube to introduce steric repulsion and act as the barriers against the interaction between nanotubes to afford good solubility in aqueous solution. The peak at ca. 1500 cm^−1^ is corresponding to the C=C aromatic of carbon nanotubes.

The I_D_/I_G_ ratios for MWNTs-COOH, MWNTS-Met and MWNTs-Met/PEG are 0.55, 0.98 and 1.07, respectively (Fig. 2b). The higher I_D_/I_G_ ratio of MWNTs-Met in comparison to MWNTs-COOH implies the successful grafting of metformin. The observed upshifting of G-band peak for MWNTs-Met/PEG in comparison to MWNTs-COOH and MWNTs-Met can be explained by the wrapping of DSPE-PEG (PEGylation) around the nanotube. The hydrophobic and van der Waals attraction between the polymers and graphite sheet increase the energy necessary for vibration to occur, which is reflected in the higher frequency of Raman peaks^25, 26^.

The ratio between intensities of the disorder-induced D and G Raman band I_D_/I_G_ is reciprocally related to the crystalline size (L_a_), where

1 $${L}_{a}(nm)=\sqrt{\frac{(4.3\pm 1.3)\times {10}^{3}}{{{E}_{l}}^{4}}{(\frac{{I}_{D}}{{I}_{G}})}^{-1}}$$

E_l_ is the excitation energy in eV^27^. Therefore, higher amount of defects correlates to higher I_D_/I_G_ ratio and reduces crystalline size. The monotonic decrease in L_a_ as shown in Table 1 indicates the introduction of modification. The reduced in L_a_ for MWNTs-Met implies the grafting of metformin due to higher I_D_/I_G_ ratio. The change in I_D_/I_G_ ratio in can be at least partly attributed to the covalent bonds between MWNTs-COOH and metformin^28^. Interestingly, it is observed only a small decrease in L_a_ for MWNTs-Met/PEG. Covalent modification typically changes the position of D-band and I_D_/I_G_ ratio whereas non-covalent modification could lead to small changes in D-band and I_D_/I_G_ ratio due to (1) D-band from disaggregation carbon particles (2) field disturbance and physical strain in graphite skeleton^25^.

**Table 1:** I_D_/I_G_ ratio and crystallite size based on Raman spectra.

	D-band	G-band	I_D_/I_G_ ratio	L_a_ (nm)
MWNTs-COOH	1320.3	1574.2	0.55	11.09 × (4.3 ± 1.3)^1/2^
MWNTs-Met	1331.7	1582.9	0.98	8.31 × (4.3 ± 1.3)^1/2^
MWNTs-Met/PEG	1331.1	1586.9	1.07	7.97 × (4.3 ± 1.3)^1/2^

The TEM images (Fig. 2c) show that the nanotubes are coated with PEG, which agree well with the FTIR and Raman results. The MWNTs-Met/PEG was soluble in water (125 µg·mL^−1^) and the dispersion remained stable with no precipitation observed after centrifugation for 15 min at 3000 rpm (inset of Fig. 2c). To further interpret the surface modification of MWNTs, ζ potential measurement was carried out (Table 2). MWNTs-COOH shows a negative potential of (−31.5 ± 0.6 mV) due to the presence of negative charged carboxyl groups. Meanwhile, MWNTs-Met shows a decreased ζ potential of −27.1 ± 0.8 mV as the result of metformin grafting which is consistent with the FTIR findings. The PEGylation leads to the shift in ζ potential closer to neutral, from −27.1 ± 0.8 mV to −17.8 ± 0.9 mV, is proof of presence of a neutral charge shielding PEG layer on the surface of nanotubes that thought to mask the surface charge^29–31^.

**Table 2:** Zeta potential (ζ) measurement in PBS.

	Zeta potential (mV)
MWNTs-COOH	−31.5 ± 0.6
MWNTs-Met	−27.1 ± 0.8
MWNTs-Met/PEG	−17.8 ± 0.9

The drug loading capability of MWNTs-Met/PEG was quantified by the thermo-gravimetric analysis (TGA) as shown in Fig. 2d. The weight loss was directly correlated to the increase of mass introduced to nanotubes at each step. Metformin experiences total weight loss at 700 °C, while the PEG decomposes at 350–400 °C. Hence, the point of near 750 °C is chosen to determine the weight ratio of metformin and PEG. As can be seen in Fig. 2d, the MWNTs, MWNTs-Met and MWNTs-Met/PEG have shown weight loss of about 7.5%, 30.0% and 45.7%, respectively. Hence, the drug loading of metformin is 22.5%, while the PEG content is 15.7%. By applying the formula (Eqn. 2) introduced by Moaseri *et al*. in quantifying the functionalization yield using TGA results, the estimated amount of metformin per gram (mmol·g^−1^) is 1.742^32–34^.

2 $$\begin{array}{c}{\rm{Drug}}\,{\rm{loading}}\,{\rm{of}}\,{\rm{metformin}}\,{\rm{for}}\,{\rm{MWNTs}}-{\rm{Met}}/\mathrm{PEG}\,({\rm{mmol}}\cdot {{\rm{g}}}^{-1})\\ \quad =\,\frac{\mathrm{Metformin}\,({\rm{mmol}})}{{\rm{weight}}\,{\rm{of}}\,{\rm{carbon}}\,\mathrm{nanotubes}\,({\rm{g}})}\end{array}$$

As shown in Fig. 2e, the temperature elevation is well correlated to the increase of power density and carbon nanotube concentration as expected. Upon NIR irradiation, nanotubes release a substantial vibrational energy due to optically stimulated electronics excitation and cause heating^35^. As can be seen in Fig. 2e, the MWNTs-Met/PEG solution quickly exceed 45 °C under 2 W·cm^−2^ NIR irradiation, indicating that the heat deposition from MWNTs-Met/PEG is sufficient to induce strong localized heating and pro-apoptosis.

**Figure 3:**
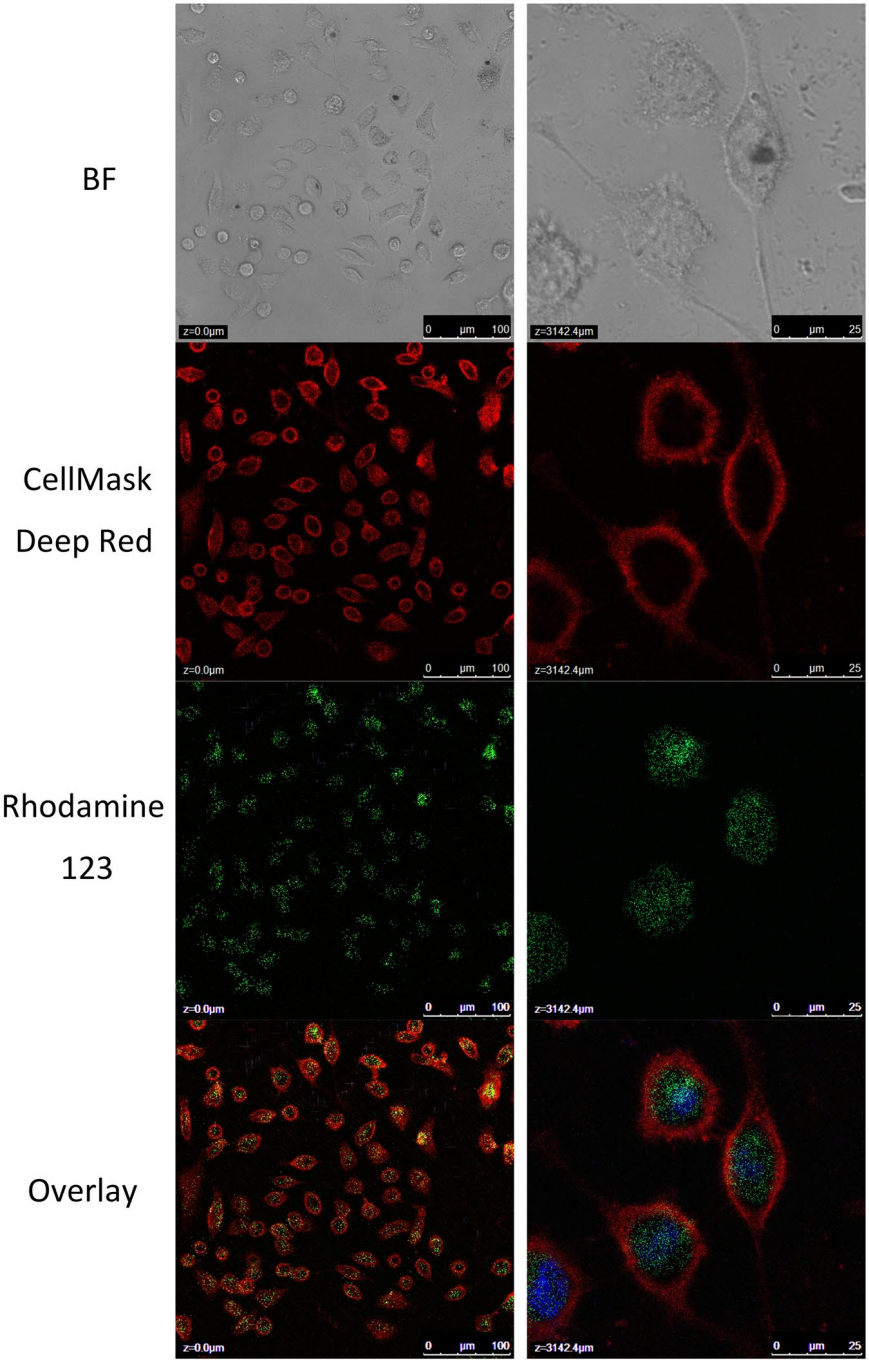
Confocal images of HepG2 cells with MWNTs-Met/PEG incubation (left) low magnification (right) high magnification. The nanotubes were found internalized and accumulated inside the cell about 3 hours after incubation.

By using confocal microscopy imaging (Fig. 3), we observed that upon exposure of MWNTs-Met/PEG to HepG2 cells, the MWNTs-Met/PEG was found to be internalized inside the cells within 3 hours after incubation. The green colour corresponded to the fluorescence labelling (Rhodamine 123) on carbon nanotubes. The cell membranes and nucleus were stained with CellMask Deep Red and DAPI, respectively. Apparently, the MWNTs-Met/PEG accumulated in the cytoplasm region. The enriched cellular uptake is correlated to the unique ability of carbon nanotube in crossing the membrane passively or translocation mechanism that is known as nanoneedle mechanism^36^.

**Figure 4:**
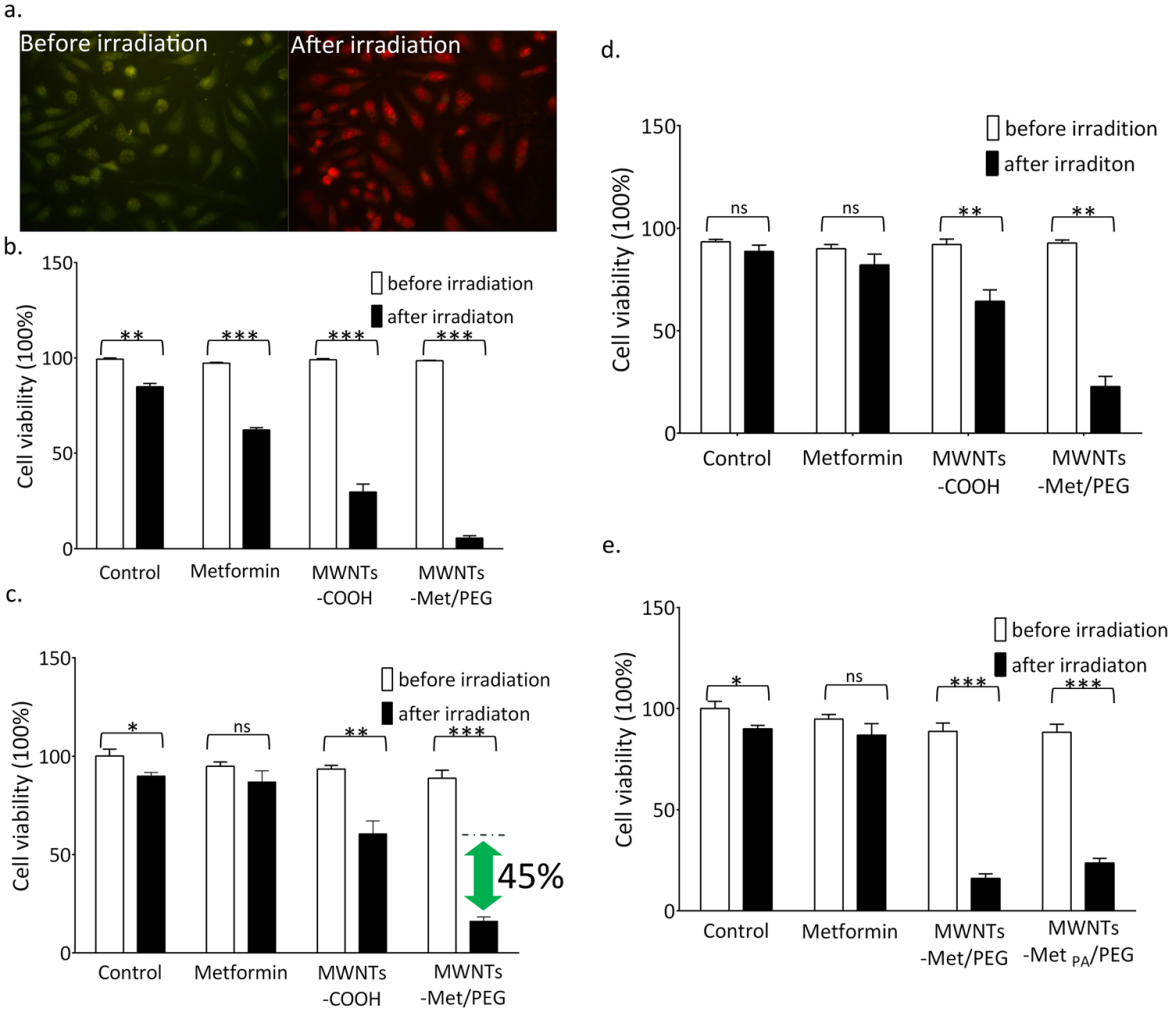
(**a**) Cell diagrams of HepG2 cells incubated with MWNTs-Met/PEG (left) before irradiation (right) after irradiation. The cell viabilities of HepG2 cells incubated with metformin (1 mM), MWNTs-COOH and MWNTs-Met/PEG under NIR irradiation were established via (**b**) AO/EB staining (**c**) MTT assay (**d**) Trypan blue exclusion assay. (**e**) Cytotoxicity determination via MTT assay for MWNTs-Met/PEG and MWNTs-Met _PA_/PEG. The MWNTs-Met/PEG contained only 35 µM of metformin as calculated from TGA. MWNTs-Met_PA_/PEG had ~72% of the drug loading of MWNTs-Met/PEG (35 uM; 1.742 mmol/g: 25 uM; 1.254 mmol/g). The NIR irradiation power density was 2 W·cm^−2^ (5 min) for **b**, and 0.8 W·cm^−2^ (8 min) for **c**,**d** and **e**, respectively. The bars are presented as the mean ± s.e.m. (Unpaired t-test, *P < 0.05, **P < 0.01, ***P < 0.0001).

The cytotoxicity of metformin, MWNTs-COOH and MWNTs-Met/PEG toward HepG2 cells under NIR irradiation (2 W·cm^−2^) were investigated using AO/EB staining. As can be seen in Fig. 4b, no apparent cytotoxicity was observed with HepG2 cells incubated with metformin (1 mM) without NIR irradiation. It is because the antiproliferation effect of metformin is well-evidenced to be prominent only under much higher doses, typically in range of several to tens of miliMolar. However, the cytotoxicity of metformin toward HepG2 cells is enhanced under NIR irradiation. The cell viability dropped to 62.2 ± 1.2% (P < 0.0001, n = 3) after being exposed to 2 W·cm^−2^ NIR irradiation for 5 mins. It is very likely the direct heating of the culturing solvent by strong NIR irradiation leads to an elevation of temperature for ~5 °C (from 37 °C to 42 °C), and subsequently induces the antiproliferation action of metformin slightly as heating would enhance the metformin’s suppression effect in cancer cells proliferation, which is in good agreement with Lee’s previous report^8^.

The cell viability of HepG2 declined to only 29.5 ± 4.2% (P < 0.0001, n = 3) with MWNTs-COOH upon NIR irradiation. The excessive localized heating inside cancer cells generated from MWNTs-COOH upon NIR irradiation was responsible to the extensive cell death, which is related to a local hyperthermia-dependent mechanism^37^. Moreover, the cancer cells are more susceptible to heat injury than normal cells due to specific biological features, reducing heat dissipating ability and lower interstitial pH^38^. Thus, it is possible to preferentially kill cancer cells rather normal cells via the localized heating.

Notably, the cell viability of HepG2 cells declined sharply to 5.7 ± 1.2% (P < 0.0001, n = 3) with MWNTs-Met/PEG upon NIR irradiation, 24% lower than that with MWNTs-COOH solely upon NIR irradiation. Besides introducing the heat injury to HepG2 cells directly which causes ~70% cells death, the extensive heat deposition from carbon nanotubes inside the cancer cells and the direct heating of the culturing solvent under 2 W·cm^−2^ NIR irradiation have also mediated a strong local hyperthermia condition and a weak overall hyperthermia condition for metformin simultaneously, which cause the additional 25% cells death.

In subsequent, the power density was attenuated to 0.8 W·cm^−2^ for 8 mins to contrast the metformin’s effect through suppressing the contribution of heat injury. MTT assay and trypan blue exclusion assay were adopted here to evaluate the viability of HepG2 cells. From the MTT assay (Fig. 4c), the cell viability of MWNTs-Met/PEG, MWNTs-COOH and metformin declined to only 16.0 ± 2.2% (P < 0.0001, n = 4), 60.6 ± 6.5% (P < 0.01, n = 4) and 86.9 ± 5.6% (P > 0.05, n = 4), respectively. The MWNTs-Met/PEG shew 45% and 71% decrease in cell viability compared to MWNTs-COOH and metformin. Whereas in the trypan blue exclusion assay (Fig. 4d), the cell viability of MWNTs-Met/PEG, MWNTs-COOH and metformin declined to only 22.7 ± 5.3% (P < 0.01, n = 3), 64.3 ± 5.51% (P < 0.01, n = 3) and 82.0 ± 5.29% (P > 0.05, n = 3), respectively. MWNTs-Met/PEG shew 41.6% and 59% decrease in cell viability compared to MWNTs-COOH and metformin. There is no statically significant difference regarding metformin solely under 0.8 W·cm^−2^ (P > 0.05). The trend is in consistent with the MTT assay. In consider the above, it is evidenced that metformin with localized heat deposition from nanotubes (MWNTs-Met/PEG) has led to significant enhancement (P < 0.01). The metformin concentration in the MWNTs-Met/PEG dispersion is only 35 µM, which 2 orders of magnitude lower than the typical effective doses (5–30 mM), for nomadic metformin in *in vitro* anti-cancer treatments according to the previous reports.

Next, we have loaded the metformin onto carbon nanotubes in two approaches, covalent bonding (MWNTs-Met/PEG) and physical absorption (MWNTs-Met_PA_/PEG). The covalent bonding has granted higher drug loading (35 uM; 1.742 mmol·g^−1^) whereas the physical absorption (25 uM; 1.254 mmol·g^−1^) has only ~72% of the drug loading of covalent bonding. Under the same condition (0.8 W·cm^−2^, 8 mins) as shown in Fig. 4e, the cell viability of MWNTs-Met/PEG and MWNTs-Met_PA_/PEG declined to 16 ± 2.2% (P < 0.0001, n = 4) and 23.6 ± 2.2% (P < 0.0001, n = 4), respectively. MWNTs-Met/PEG show 7.6% decrease in cell viability compared to MWNTs-Met_PA_/PEG. Apparently, the 7.6% decrement is attributed to the higher content of metformin grafted, evidencing the contribution of metformin and synergism effect.

Conventional dosing of metformin is sufficient to result in an indirect cytostatic effect on certain tumours that thrive in hyperinsulinemic and hyperglycemic environment through the suppression of liver gluconeogenesis due to hepatocyte energy stress, even if metformin does not accumulate in neoplastic tissue. On contrary, if adequate drug levels are achieved in cancer cells that have intact mechanisms to cope with energetic stress, it will result in reduced cellular energy consumption via modulating the signalling pathways. It is expected to have important antiproliferation effect^18^.

To achieve sufficient drug level in neoplastic tissue for prominent antiproliferation effect, the metformin concentration used experimentally has far exceeded than those achievable clinically. The extreme dose is rarely in clinical setting and known to associate fatal adverse effect. Thus, the results obtained cannot be considered as an approximation of clinical use and directly extrapolated to potential effect in clinical trial^39^. It is paramount to achieve sufficient drug levels in neoplastic tissue to allow the clinical evaluation yet retains the administrated metformin concentration at the level of conventional antidiabetic dose.

As previously mentioned, conventional hyperthermia with prolongs heating time up to 6 h has result in observable enhancement in the metformin antiproliferation action even at the conventional diabetic dose^8^. Lee *et al*. concluded the heat-induced enhancement of metformin cytotoxicity is mediated by potentiation of AMPK activation. Hyperthermia has been demonstrated to activate AMPK and suppresses mTOR activity. Owing to the excellent photothermal and translocation behaviour of carbon nanotubes, we have introduced a short and highly localized heating to intensify the antiproliferation action of metformin at the conventional antidiabetic dose. The outcome is comparable to the condition of aggressive dosing in metformin monotherapy. The localized heating has the superiority in spatial confinement over conventional hyperthermia. Therefore, the temperature elevation could exceeds the safe upper limits of hyperthermia (42–43 °C) with minimal collateral tissue damage^40^. The significant synergistic effect observed in elevated temperature may possible due to AMPK activation and drug-heat interaction^8, 41^. In our model, the low dose with high spatial heating confinement minimizes the adverse effect and facilitates the possible use in clinical.

## Conclusion

Although we are unable to conclude the mechanism for the enhancement effect under localized heating at the current stage, we have demonstrated the promise of using highly localized hyperthermia-like condition from carbon nanotube to intensify the efficacy of metformin and addressed the dilemma of aggressive dosing for the potential cancer therapies.

## Experimental Section

### Preparation and characterization of MWNT-Met/PEG

35 mg of MWNTs were mildly oxidized by mixture of nitric acid and sulphuric acid (1:3 v/v) in sonication bath for 120 min at 50 °C. Then the mixture was filtered and washed to obtain filter cake of carboxylated MWNTs (MWNTs-COOH). MWNTs-COOH was diluted in 50 mL Morpholinoethanesulfonic acid Monohydrate buffer. 180 µl of EDAC and 0.2 g of N-Hydroxysuccinimide (NHS) were added into dispersion, followed by rhodamine 123 and 0.165 g of metformin hydrochloride in 4 hours later. The dispersion was then stirred overnight for 24 h under room temperature of 25 °C. The dispersion was filtered and washed to obtain MWNTs-Met filter cake. The filter cake was then re-dispersed in 30 ml of 0.4 g·L^−1^ DSPE-PEG. The dispersion was placed in sonication bath for 30 min at 50 °C. Lastly, the dispersion was filtered and washed to obtain the final form of MWNTs-Met/PEG and dried overnights at 60 °C. For preparation of MWNTs-Met_PA_/PEG, the method was identical to MWNTs-Met/PEG except that EDAC, NHS and rhodamine 123 were not added. IR spectra were conducted by using VERTEX 70 FT-IR spectrometer, Bruker Optics. Raman spectra were obtained by using LabRAM HR 800, Horiba JOBIN YVON (633 nm). The zeta potential measurements were conducted by using zeta potential analyser (Zetasizer Nano ZX90, Malvern). Visual characterization was conducted by using TEM JEM-2100, JEOL. TGA curves were conducted by using TGA/DSC, Mettler Toledo.

### *Ex Vitro* Measurement of heating of MWNTs-Met/PEG solution by NIR radiation

The MWNTs-Met/PEG solution was irradiated by 808 nm NIR laser at 0.5 W·cm^−2^ with tuneable power density up to 2.0 W·cm^−2^. The laser beam was reflected with dielectric lens several times so that the beam was directed perpendicularly to the cell at direction of Z-axis. The temperature elevation was measured via thermocouple and data logging system from Omega. Care was taken to shelter the thermocouple from direct exposure to laser beam path to minimize any direct heating from laser.

### Cell culture and cellular incubation with MWNTs-Met/PEG

The HepG2 cells were cultured in DMEM supplemented with 10% FBS and 1% penicillin-streptomycin in 24-well plates. The cells were seeded for 18/24 h before the incubation with MWNTs-Met/PEG at final concentration of 20 µg·mL^−1^. The incubation was carried out at 37 °C and 5% CO^2^ atmosphere.

### *In vitro* photothermal therapy of HepG2 cells

The HepG2 cells with and without nanotubes incubation were subject to 808 nm NIR irradiation. To avoid contamination, the well plates cover was sealed during whole process of laser irradiation. The laser beam was reflected with 808 nm dielectric lens several times so that the beam was directed perpendicularly to well plates at direction of Z-axis. The exposure time was 5 min with power density of 2.0 W·cm^−2^ and 8 min of 0.8 W·cm^−2^, respectively. The beam size was ~1 cm, nearly fully covering the area of individual column of 24-well plates. To mimic the biological environment, the well plate was heated to 37 ± 1 °C and maintained over laser irradiation process by a custom-made temperature control system.

### Acridine orange (AO) and ethidium bromide (EB) fluorescent assay for cell death

AO and EB staining was performed as described by Spector *et al*.^42^. HepG2 cells were cultured separately in 24-well plates and treated with the complex (metformin, MWNTs-COOH and MWNTs-Met/PEG) for 48 h, where PBS (10 mM) was used as solvent control. After laser excitation for 5 min (808 nm, 2.0 W·cm^−2^), the treated and untreated cells were incubated with acridine orange and ethidium bromide solution (1 part of 100 μg·mL^−1^ each of acridine orange and ethidium bromide in PBS) and examined in a fluorescent microscope (Carl Zeiss, Jena, Germany) using a UV filter (450–490 nm). Three hundred cells per sample were counted, in triplicate, for each time point and scored as viable or dead, and if dead whether by apoptosis or necrosis as judged from the nuclear morphology and cytoplasmic organization.

### MTT cell proliferation assay

The HepG2 cells were suspended in DMEM media and plated at a density of 5 × 10^3^ cells/well into a 96-well culture dish. After 24 h, the medium was replaced with DMEM in the presence or absence of complex (metformin, MWNTs-COOH, MWNTs-Met/PEG and MWNTs-Met_PA_/PEG) for 24 h, followed by laser excitation for 8 min (808 nm, 0.8 W·cm^−2^). The viability of HepG2 cells were detected by a 3-(4,5-dimethylthiazol-2-yl)−2,5-diphenyltetrazolium bromide (MTT) cell proliferation assay kit.

### Trypan blue exclusion assay

The HepG2 cells were suspended in DMEM media and plated at a density of 2 × 10^4^ cells/well into a 24-well culture dish. After 24 h, the medium was replaced with DMEM in the presence or absence of complex (metformin, MWNTs-COOH and MWNTs-Met/PEG) for 24 h, followed by laser excitation for 8 min (808 nm, 0.8 W·cm^−2^). The viability of HepG2 cells were detected by trypan blue and direct cell counting using hemacytometer.

### Confocal imaging

HepG2 cells were cultured in DMEM medium supplemented with 10% fetal bovine serum and penicillin-streptomycin 1%. The incubations of MWNTs-Met/PEG with HepG2 cells were carried out on 3.5 mm plates, with the cells having been seeded for 24 hours before MWNTs-Met/PEG was added. The incubations were carried out at 37 °C in 5% CO^2^ for 24 hours. After incubation, the CellMask Deep Red was added and the cells were washed three times with phosphate-buffered solution. The cells were imaged by a scanning confocal fluorescence microscope.

### Statistics

All data are presented as the mean ± standard error of the mean (s.e.m.). Data presented were analysed with unpaired t-tests. Statistical analysis was performed in Graphpad Prism 6.0 and P < 0.05 was considered as statistical significant in all analyses.
